# Control and Entropy Analysis of Corner Flow Separation in a Compressor Cascade Using Streamwise Grooves

**DOI:** 10.3390/e21100928

**Published:** 2019-09-24

**Authors:** Weilin Yi, Lucheng Ji

**Affiliations:** 1 School of Mechanical Engineering, Beijing Institute of Technology, Beijing 100811, China; 2 School of Aerospace Engineering, Beijing Institute of Technology, Beijing 100811, China; jilc_bit@126.com

**Keywords:** flow separation, streamwise grooves, cascade, secondary flow, entropy analysis

## Abstract

Flow separation, which often occurs at the junction of blades and endwalls and seriously limits the aerodynamic performance of turbomachinery, is caused mainly by the boundary layer mixing on the blades and endwall surfaces and the transverse secondary flow. Focusing on a linear diffusion cascade with 42° turning angle, the transverse secondary flow is found to be the dominant factor for flow separation, based on detailed analysis. Therefore, controlling the secondary flow to reduce the flow separation is very important. Based on the investigations, the flow separation can be controlled by cutting off the secondary flow. Therefore, nine kinds of streamwise grooves were designed and analyzed herein. Grooves at the endwall substantially inhibited the transverse secondary flow, but the flow structure varied over different spans. An optimum combination of groove width and height was identified, with the height being more important. A detailed flow analysis of the best scheme (with a smaller width and moderate height) was conducted. The loss reduction mechanism was obtained by 3D flow field and entropy analysis. This configuration can reduce the corner zone separation effect, energy loss coefficient, and flow loss.

## 1. Introduction

Turbomachinery are very commonly used in turbochargers, gas turbines, aero engines, and so on. The performance of the turbomachinery, mainly determined by flow field structures, has a critical effect on these important devices. Thus, improving aerodynamic performance is consistent pursuit of turbomachinery design. In turbomachinery, flow separations often occur near the junctions between the blades and endwalls, which are the major loss sources. Gbadebo [1] pointed out that flow separation in corner regions in turbomachinery without tip clearance appears to be universal. Three-dimensional separation often occurs not only by mixing of the boundary layers on the blades and endwall surfaces, but also by transverse secondary flow, which is mainly generated by the pressure difference between the pressure side and suction side of the blades in the corner zones [2,3] and has a dominant effect on aerodynamic performance. In compressors, the effects are more serious and can cause stalling, usually because of the strong adverse pressure gradient. For these reasons, modern compressor blades diffuse the flow efficiently only in the range 20%–80% of the span height [2]. Additionally, a large amount of research has shown that the losses in the corners account for 30% of the total losses in these passages [4]. Based on these findings, many investigations [5,6,7,8,9], have focused on the flow mechanism of corner zone flow. Wang [10] also used entropy analysis to evaluate the loss mechanism in a compressor cascade based on numerical simulation results.

Many different flow control strategies have been put forward. Three-dimensional blade designs incorporating lean, bow, and sweep are important for reducing endwall losses and improving aerodynamic loading levels. Breugelans [11] investigated the influence of lean on linear cascades. Weingold [12] and Fischer [13] implemented bow into the stator, reducing the flow separation and increasing the efficiency. Tweedt [14] and Wadia.A.R [15] implemented a design combining bow and sweep in a multistage compressor to improve the performance. However, some researchers, for example, Gummer [16] and Barankiewicz [17], have found that corner flow separation cannot be fully defined by three-dimensional blade design. Therefore, another specific method for controlling corner flow separation with a nonsymmetrical endwall has been developed and investigated. This technique can control the secondary flow from the pressure side to the suction side and reduce losses. Dorfner [18] investigated the application of this technique in the third stage stator in a three-stage compressor. Rolls-Royce [19] incorporated a nonsymmetrical endwall into a six-stage compressor. Though the performance of the compressor was improved, the effectiveness was not as obvious as that in a turbine. In addition, the boundary layer suction technique [20,21] and plasma excitation technique [22,23] have also been investigated by many researchers. However, in general, the addition of devices, complex fabrication, and off-design adaptations lessen the practicability and necessitate long development times for these techniques.

In addition to these measures, many researchers have found that some special kinds of rough surfaces can reduce wall drag and improve the flow structure near the wall [24,25]. For examples, groove, riblet, and fence designs have been suggested in some studies to control the flow structure near the wall [26,27,28]. Some useful attempts have also been carried out in turbomachinery. The flow in turbines is relatively good and easy to control, so some initial studies about grooves were carried out in turbines design. Zhang [29,30] designed different kinds of riblets with streamwise grooves to change the hub wall shape in an axial turbine cascade. The results showed that the horse-shoe vortex near the pressure side can be reduced. Khader [31] investigated the effect of the riblets/grooves on the performance of the radial turbine, and found that these structures reduced the cross secondary flow and the shear force near the wall. Related studies on the complex flow filed under a negative gradient flow in compressors are rare. Oehlert [32] added the V-shaped riblet togenerated grooves on the blade surface of the compressor cascade and reduce the total pressure loss by 3.6%. However, Alexander [33] carried out more detailed researches and obtained different results. Two kinds of rough surface, rolling and coating surface, were added to the blade surface of the compressor cascade. The results showed that the drag loss was not reduced. In contrast to the above studies which added grooves to the blade surface, Dorfner [34] first added grooves on the hub wall of the compressor cascade. A single groove combined with a nonaxisymmetric endwall was designed as an aerodynamic separator to control the secondary cross flow and suppress the corner separation. 

In summary, the transverse secondary flow near the hub wall is an important cause of flow separation in the corner zone, and this separation is not controlled very well in compressors. Grooves or riblets can be added directly to the endwall to control the secondary flow and possibly reduce the flow separation, but the related research is still insufficient, especially relating to compressors. Thus, in this paper, we designed a compressor cascade and analyzed the loss mechanisms. Nine kinds of multiple grooves along the streamwise direction were added to the hub wall and investigated to determine whether the performance could be improved.

## 2. Geometry and Flow Analysis of the Baseline Cascade

### 2.1. Geometrical Descriptions

**Figure 1:**
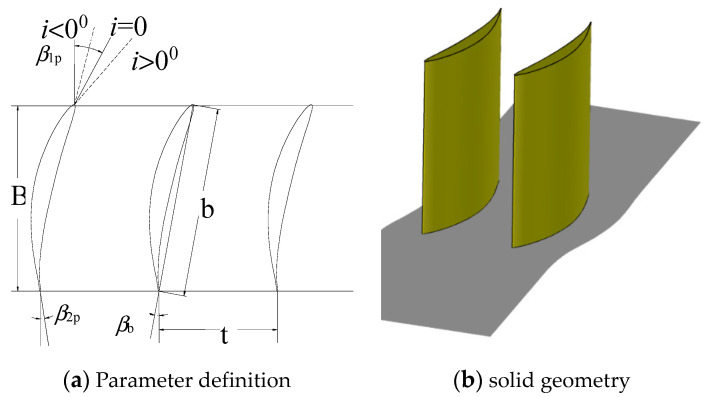
Sketch of the cascade.

The research objective in the present paper is a linear compressor cascade. The linear straight cascade used in this paper was stacked with an airfoil scaled from NACA65. The airfoil turn angle was 42°. The main geometrical parameters are shown in Table 1. The airfoil sketch and solid model are shown in Figure 1. More detail information about this cascade can be found in References [35,36]. 

### 2.2. Computational Solver

Nowadays, CFD methods are widely used for turbomachinery flow analysis. The calculation results are dependent on the chosen software, turbulence models, and solver settings. The CFD code used in this study was the Numeca Fine/Turbo solver, which has been widely used in many academic institutes and industry companies [37,38]. The steady Reynolds-averaged Navier–Stokes equation was carried out in the present paper. This equation numerical solver is density-based and uses the four-stage Runge–Kutta numerical technique with Jameson’s artificial dissipation and implicit numerical smoothing. The spatial discretization was selected as a second-order, cell-centered explicit finite volume scheme. For turbulence closure, the software employed a variety of turbulence models. Based on the recommendations of Numeca help and Reference [39], the Spalart–Allmaras turbulence model was used for the fine capture of near-wall flow structures.

### 2.3. Computational Domain and Boundary Conditions

**Figure 2:**
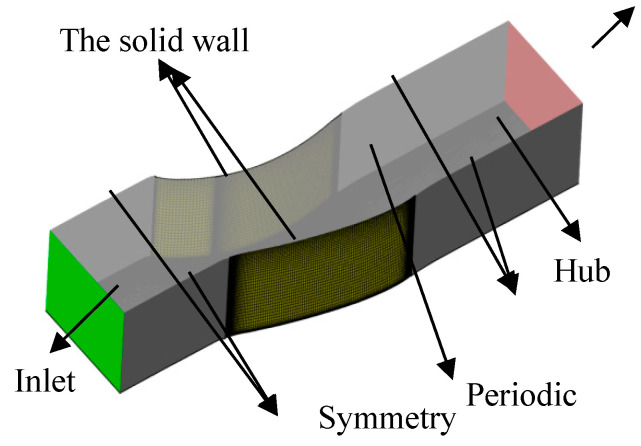
Computational domain and boundary type.

**Figure 3:**
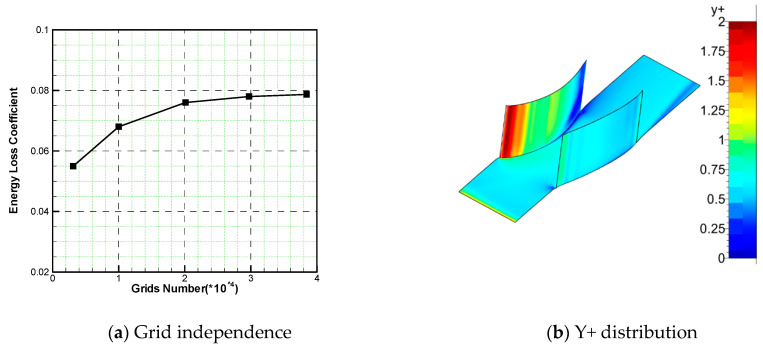
Grid independence verification and Y+ distribution.

The comulational domain is shown in Figure 2. The mesh generation was finished using Numeca Autogrid software, which is a widely acknowledged tool for turbomachinery mesh generation. Multi-block structure topology was applied. In order to save computational time, the computional domain was selected as the half-span blade passage because of the symmetry of flow status from hub to shroud endwall. For the convenience of generating meshes for different schemes, the mesh topology used was H type. In order to verify grid independence, five sets of grids were generated in this paper. The comparison of the total pressure loss coefficient at the outlet with different meshes is shown in Figure 3. It can be seen from the picture that the simulation results above 2 million grids agreed in both the trend and the value. We concluded that the 2 million grids accurately simulated the details of the flow field, and produced credible performance data. In order to describe the flow structure in more detail, the following numerical simulation was all based on 3.85 million grids. The maximum y^+^ was less than 2, as shown in Figure 3b. 

We used the entropy and energy loss coefficient to compare the changes for different kinds of conditions. The definition of entropy [40] and the energy loss efficiency are listed as follows:

(1) $$s=c_{v}\pi \ln\frac{\left(p/p_{inlet}\right)}{{\left(\rho /\rho_{inlet}\right)}^{k}}$$

(2) $$\bar{\xi }=\frac{{\left(p/P^{*}\right)}^{\frac{k-1}{k}}-{\left(p/P_{0}^{*}\right)}^{\frac{k-1}{k}}}{1-{\left(p/P_{0}^{*}\right)}^{\frac{k-1}{k}}}$$

where $P_{0}^{*}$, p, ρ, $P_{}^{*}$, and κ are the inlet total pressure, local static pressure, local total pressure, and specific heat ratio, respectively.

**Figure 4:**
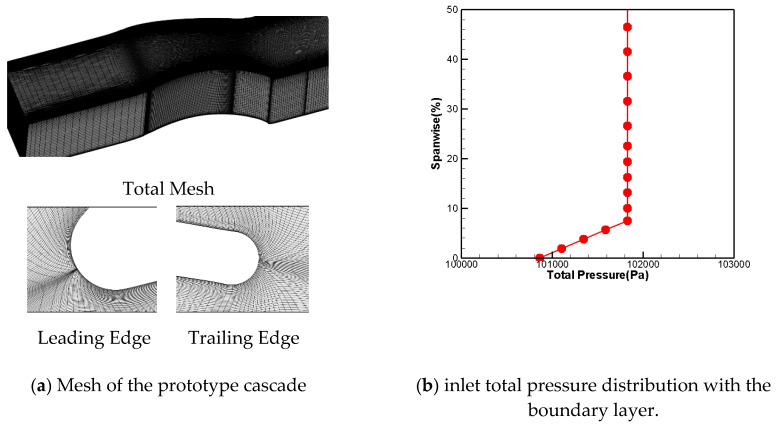
Single passage of the cascade.

As shown in Figure 2, the inlet of the domain was taken upstream of the blade and normal to the axis at a 50% chord length before the leading edge of the cascade. Total conditions based on the total pressure $P_{0}^{*}$ with the boundary layer, total temperature $T_{0}^{*}$, and airflow angle were imposed. The thickness of the inlet boundary layer was 8%, and the profile for the total pressure is shown in Figure 4b. The total temperature was a constant. To investigate the performance at different incident angles, the simulations were carried out with angles of 0° and +5°.

The outlet of the domain was taken downstream of the blade and normal to the axis at a 100% chord length after the trailing edge of the cascade. An average static pressure was prescribed at the outlet. For the walls, no-slip and adiabatic conditions were defined.

### 2.4. Computational Results

**Figure 5:**
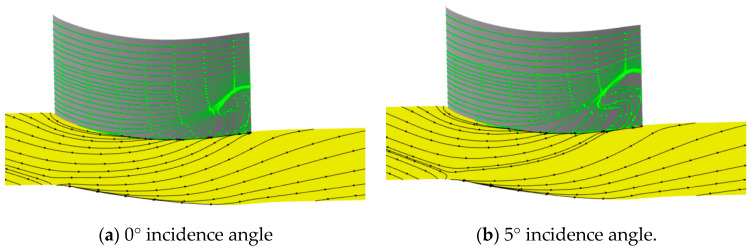
Streamlines at the suction side and hub wall.

We first analyzed the internal flow details and performance parameters for the prototype cascade. Figure 5 shows the streamlines at the suction side and the endwall for the 0° and +5° incidence angles. Obvious flow separation appeared at the corner region near the trailing edge of the cascade. When the incidence angle was 0°, the separation zone started from the 50% chord length position in the passage and occupied approximately 25% of the span height from the hub. When the incidence angle was 5°, the separation zone started slightly earlier and occupied approximately 30% of the span height from the hub. There were obvious transverse secondary flows at the hub, but the core of the separation zone was not very near the endwall.

**Figure 6:**
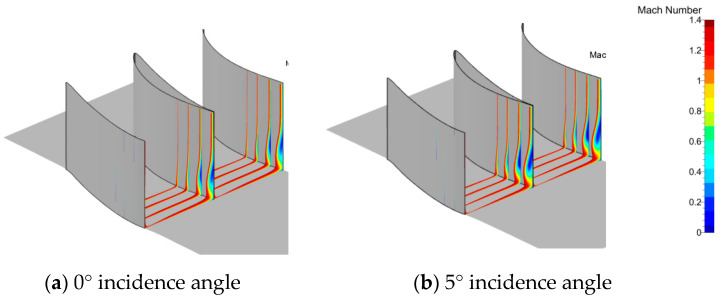
Mach number distributions in different sections. 10%, 20%, 30% chord length from the trailing edge).

**Figure 7:**
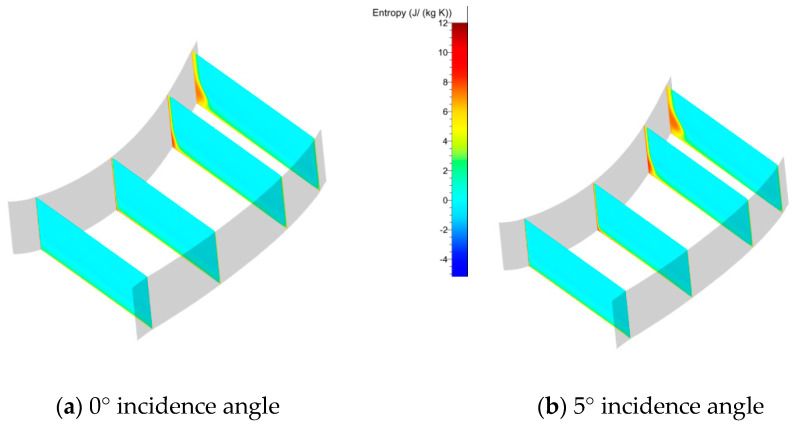
Entropy analysis at different incidence angles.

Figure 6 shows the Mach number distribution at different sections vertical to the axis. The distance to the trailing edge was 30%, 20%, 10%, and 0% of the chord from left to right. The low-velocity zone was not very close to the hub and gradually increased in the spanwise direction from left to right. The core of the low-velocity zone was located at 10% of the spanwise height when the section reached the trailing edge. Moreover, the height of the core position increased with increased incidence angle. Why was the core of the separation zone so far away from the hub wall? One guess is that the turn angle of the cascade was only 42°, thus avoiding serious separation in the blade surface, so the transverse secondary flow pushed the low-energy fluid in the boundary layer to the suction side and rolled it to a greater height in the span from left to right. This meant that the secondary flow was the main reason for the flow separation in the corner zone. It was convenient to use entropy to describe the degree of loss. From the entropy analysis in Figure 7, the high-loss zone in the present cascade was located in the suction-hub corner near the trailing edge. These trends intensified with increasing incidence angle. The reason for this was the interaction between the boundary layer separation flow and passage vortex. It can be seen that the extent of loss was relative to the separation zone size. However, the size of the separation was directly related to secondary flow. Thus, if the secondary flow can be controlled, the corner zone separation can be reduced and the extent of loss can be decreased. 

## 3. Design of the Streamwise Grooves at the Hub Wall

Next, we wished to cut off the transverse secondary flow from the pressure to the suction side. Based on the conclusions outlined in the introduction section, we added grooves to realize the same effect, aiming to determine how long the grooves should be, how many grooves should be incorporated, and their optimal heights and widths.

**Figure 8:**
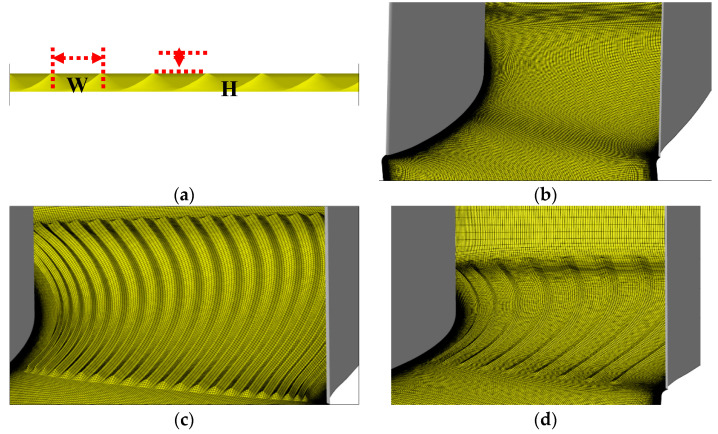
Geometry model of the streamwise grooves. (**a**) Design parameters of the streamwise grooves at the hub wall; (**b**) mesh of baseline cascade; (**c**) mesh of Case 3; (**d**) mesh of Case 9.

We designed nine multiple-groove schemes. As shown in Figure 8, several streamwise grooves were configured broadly parallel with the pressure side and the suction side. We developed different schemes with different start positions, end positions, width W, and height H.

In order to guarantee the mesh consistence of the baseline and grooved cascades, the authors developed a Fortran code to adjust the original structural mesh generated by Numeca AutoGrid Software. Near the grooves, the mesh lines were moved smoothly to form high quality meshes for nine groove schemes, as shown in Figure 8. The geometric parameters of the nine groove schemes are shown in Table 2.

## 4. Results Analysis 

**Figure 9:**
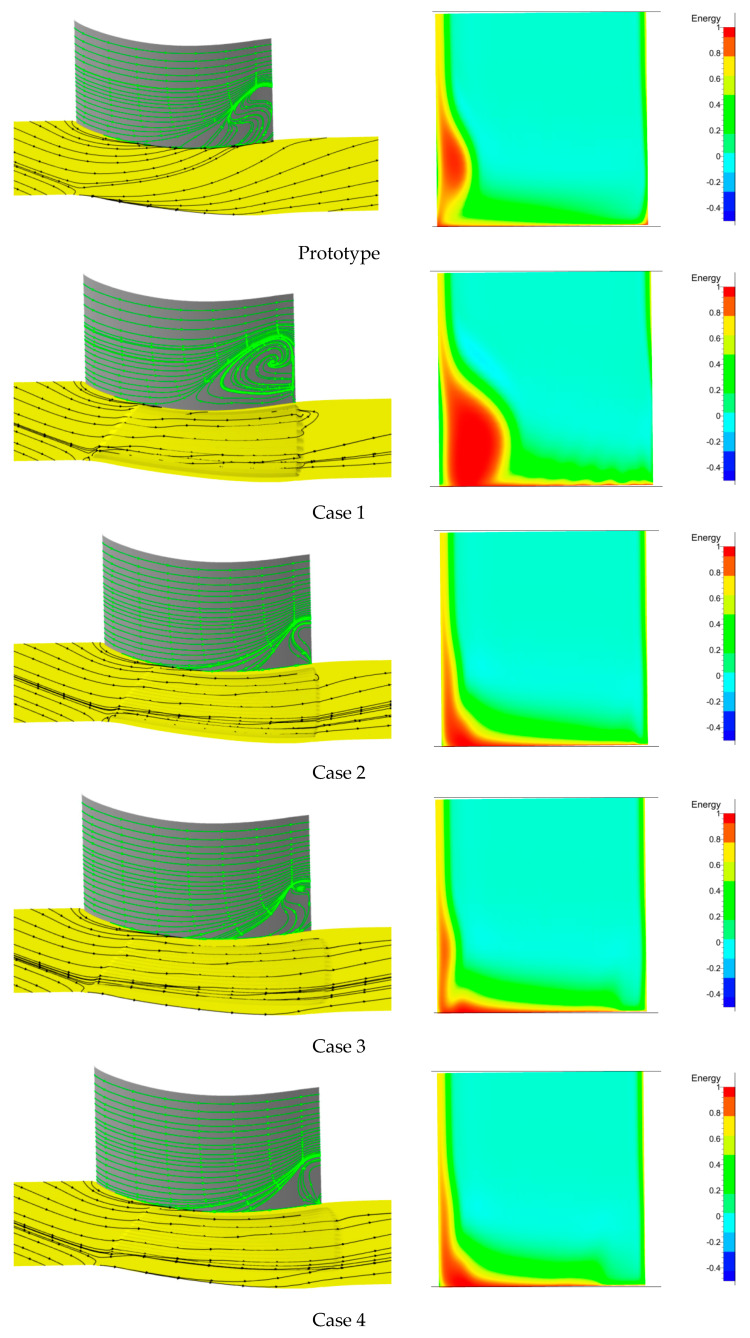

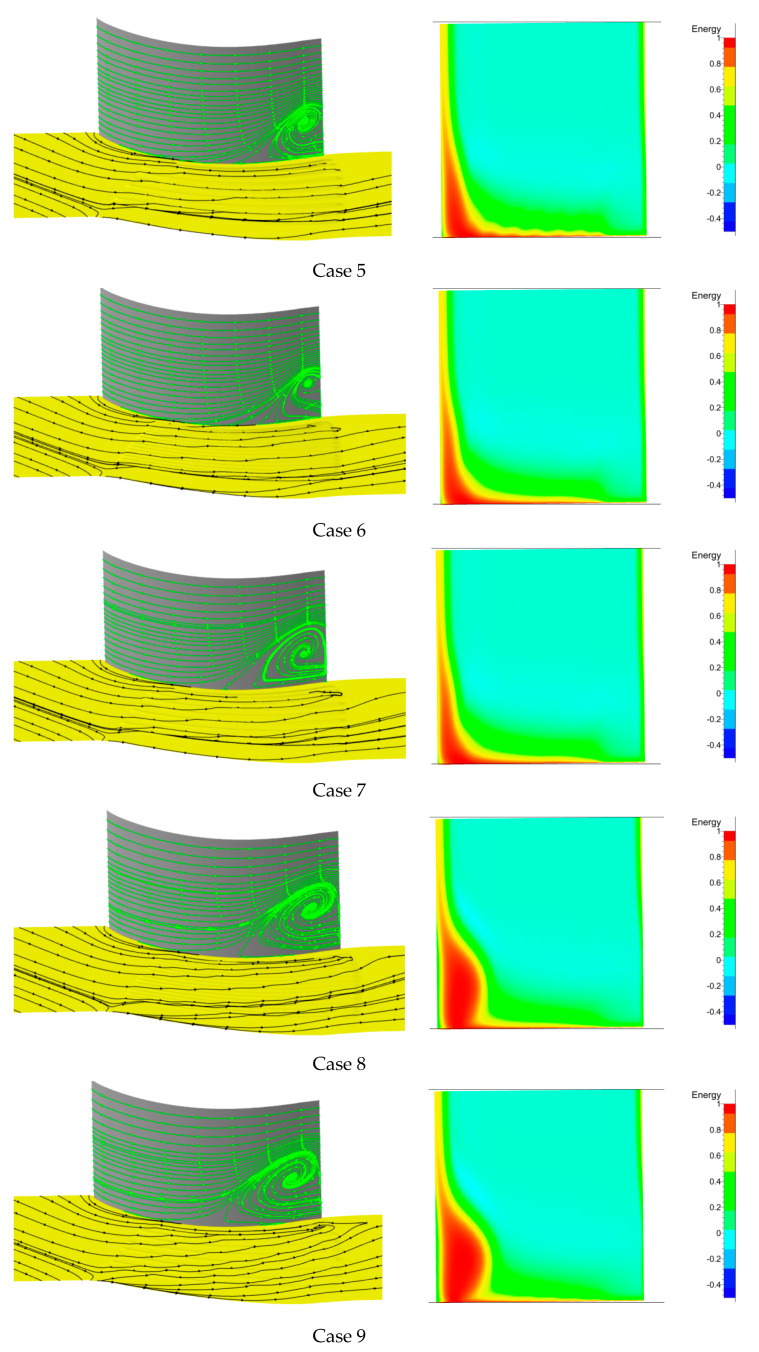
Comparison of the prototype and groove cases for a +5° incidence angle. (**left**) Streamlines; (**right**) energy loss coefficient of the cascade outlet.

Details of the flow field comparison between the prototype cascade and grooved cascade are shown in Figure 9 only under the +5° incidence angle condition. In any case, the +5° incidence angle is a typical operational configuration and possesses representative characteristics. The effect of the different groove schemes, including their strength, shape, and scope, on the streamlines and separation are large.

In the present paper, the start and end positions of the different grooves schemes were the same; only the widths and heights were different. The widths and heights were less than the inlet boundary layer thickness. According to the machining and mesh quality, we designed groove structures with W < 5.5 and H < 2.5. Considering simulation time, the nine structures shown in Table 2 were selected. 

We firstly analyzed the change in the secondary flow and the separation zone on the wall surface of the cascade and the change in the energy loss coefficient in the outlet section of the cascade. Based on Figure 9, the transverse secondary flow in the hub wall was clearly reduced compared with the prototype cascade. The streamlines generally followed the directions of the grooves. However, note that the flow field near the hub wall had different backflow zones in the different groove schemes. The size of the backflow zone was determined mainly by the distance between the suction side and the nearest groove.

Secondly, although the transverse secondary flow was reduced in the groove schemes, the separation zones on the suction surface had different ranges and strengths compared with those of the prototype cascade, which was related to both the width W and the height H. From the above results, a qualitative conclusion was obtained that when H was small and W was big, as shown in Cases 8 and 9, the separation zone was increased compared with that of the prototype cascade.

**Figure 10:**
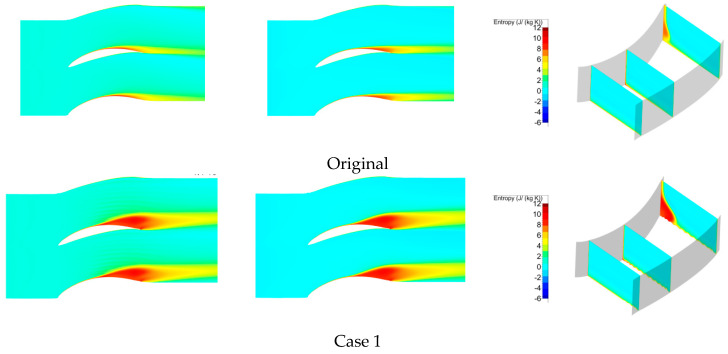

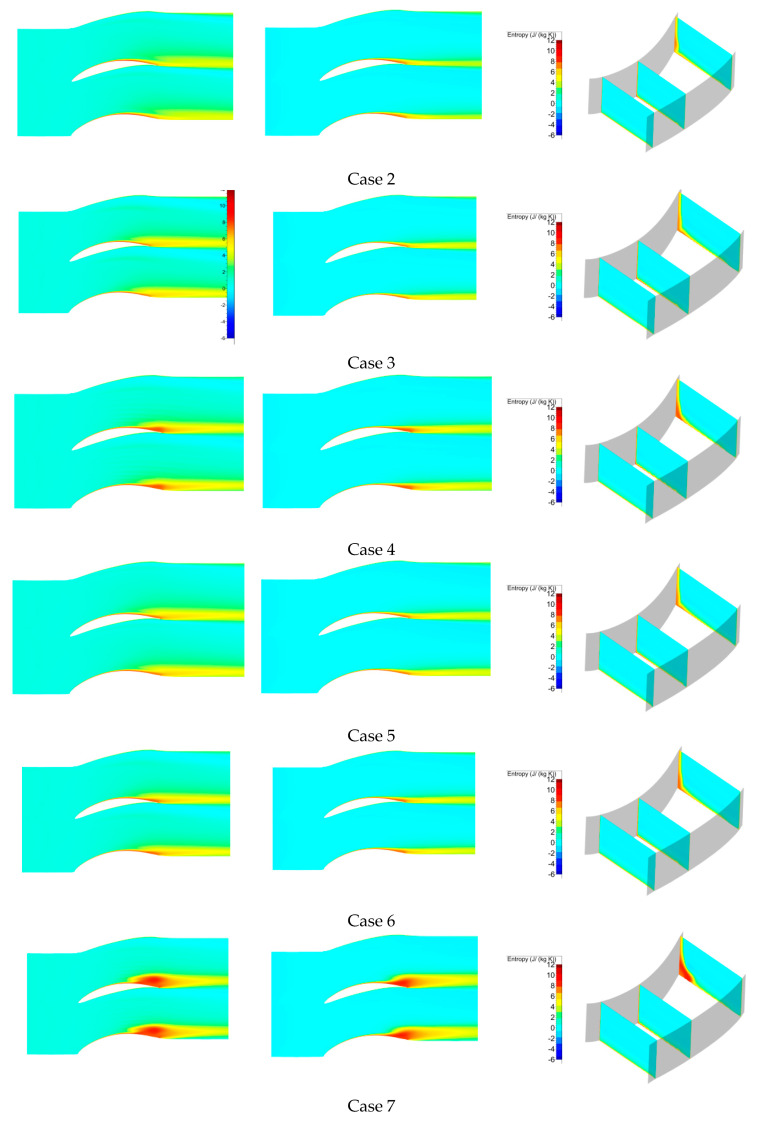

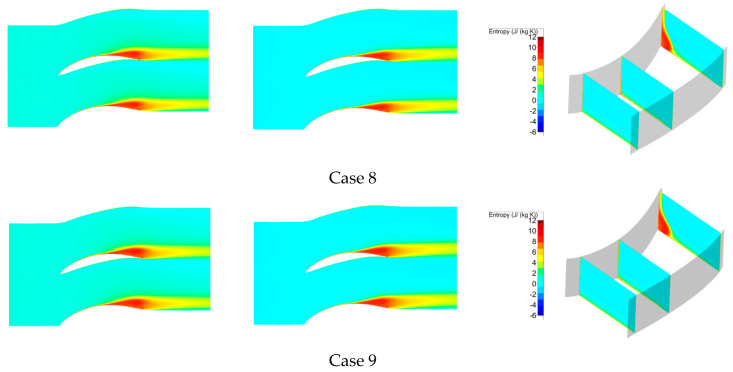
Entropy analysis of the different spans for a 5° incidence angle. (**left**) 5% span; (**middle**) 10% span; (**right**) streamwise.

Based on the entropy generation analysis, the loss status was obtained more detail. For example, from the entropy analyses for Cases 8 and 9 in Figure 10, the high-entropy zone was enlarged near the suction side at the 5% and 10% spans. With increasing H, as shown in Cases 2–7, the separation zone was decreased. However, in Case 1 (with the highest H), the separation zone increased again. Therefore, the height H produced a peak value for the losses of the cascade corner zone. The entropy analyses of loss characterization for Cases 2 to 7 also demonstrated this. For a constant height H, for example, Cases 2–5, the weight W also had a peak value for the losses of the cascade corner zone. However, the effect of the width W was not notable, in contrast to that of the height H. The energy loss coefficient is shown on the right side of Figure 9. We found a high-energy-loss zone related to the separation zone, and the distributions were different when W and H had different values. Therefore, we know that there is an optimum combination of width and height; furthermore, the height is more important than the width.

**Figure 11:**
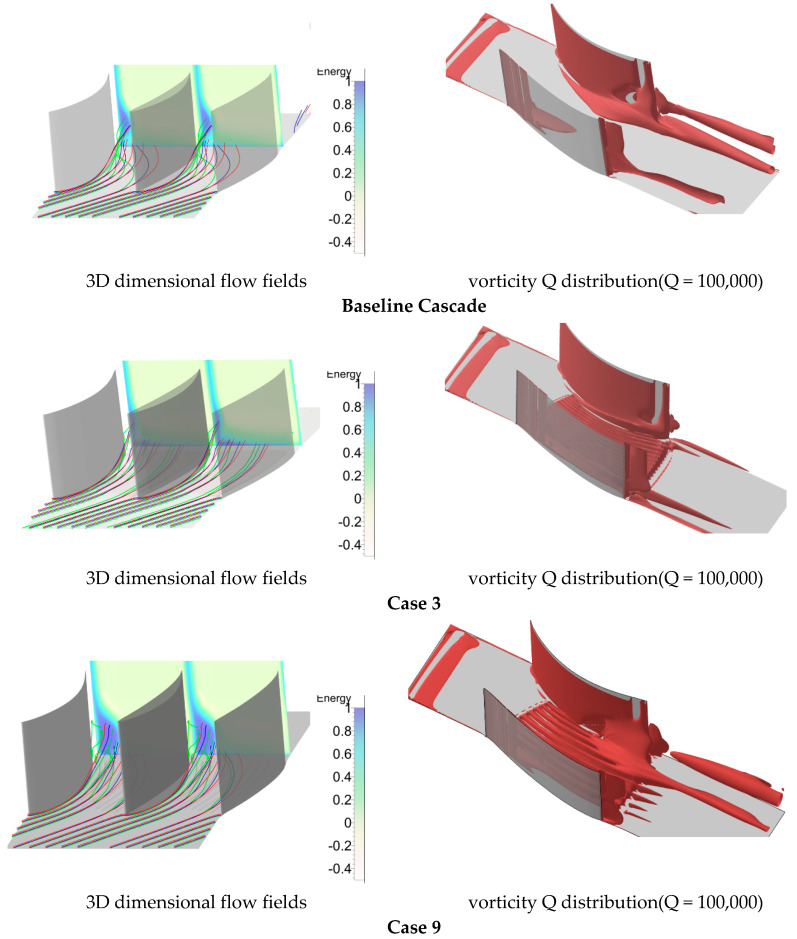
3D dimensional flow fields and vorticity Q distribution (Q = 100,000).

Since the streamwise grooves weakened the transverse secondary flow, why was the separation zone not always suppressed? How do the parameters W and H affect the flow field in different flow structures? We answered these questions based on several typical cases, namely the original case, the worst case (Case 1), and the best case (Case 3). Figure 11 shows the three-dimensional flow fields and the vortex visualized with Q = 100,000. For the original cascade, the 3D streamlines were obviously turned under the pressure difference between pressure and suction sides. Thus, the fluids were rolling up to form the separation vortex. Based on the vorticity Q = 100,000, the vortex was apparent near the suction-hub corner zone and propagated downstream. Compared with the original cascade, the 3D streamlines of Case 3 were smoother and the loss was smaller. This was also seen from the vorticity Q, shown on the right side. For Case 9, the energy loss was very high. Although the 3D streamlines were not seriously turned, the rolling up tendency was more serious for the reverse flow analyzed in Figure 9. Thus, the separation vortex strength was increased, as shown in Figure 11.

**Figure 12:**
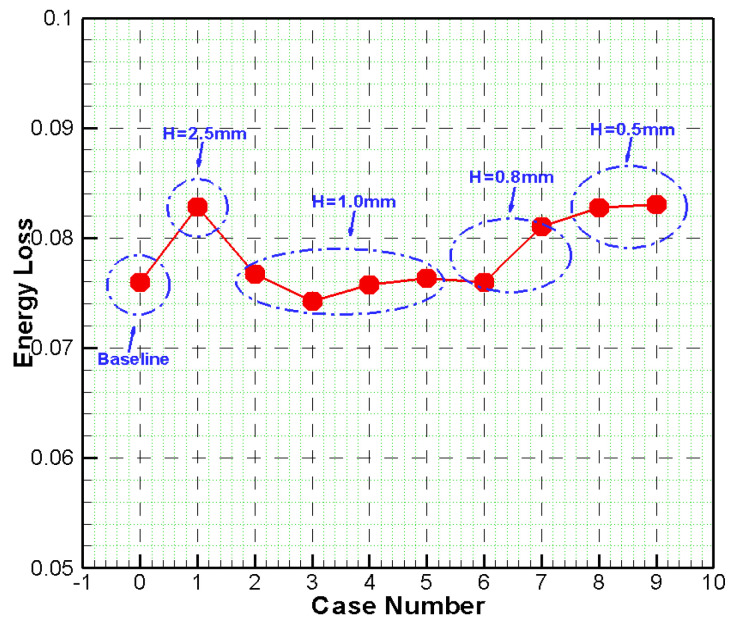
Energy Loss Distribution with Different Cases.

The quantitative assessment of energy loss with different cases is shown in Figure 12. It can be seen that there is an obviously feature for optimum grooves heights with h = 1.0 mm. In the range of 0.5mm to 2.5mm for height, when the height is lower or higher, the loss is higher than in h = 1.0 mm. For the same height, no obviously trend is for groove width. So based on the above analysis, we can conclude that the height is more important for loss reduce.

## 5. Conclusions

Controlling the flow separation at the corner zone is the key step in increasing the aerodynamic load in compressors. Moreover, the transverse secondary flow is the dominant factor inhibiting the loading increase and leading to flow separation at the corner zone in the cascades used in the present paper. Thus, based on a detailed numerical analysis of the prototype cascade, nine streamwise groove schemes at the hub wall were designed, and the internal flow details and entropy generation were analyzed.

The streamwise grooves at the endwall are found to substantially inhibit the transverse secondary flow and to smooth the flow along the flow direction. However, the flow structures are different at different spans, and poor design schemes cause backflow and increase the separation near the suction side-hub corner zone. There is an optimum combination of groove width and height, and the height is more important than the width.

Among all the examples in the paper, the performance of Case 3, with a relatively small width and moderate height, is the best (width W = 2.0mm and height H = 1.0mm). This configuration increases the 3D streamlines along the groove direction and decreases the vortex strength (as shown in vorticity Q). Thus, the separation zone scope at the corner zone is weakened and the entropy generation and the flow losses are reduced.

The present study is restricted to a plane diffuser cascade with inlet Mach number 0.2; the conclusion cannot necessarily be applied directly to a higher inlet Mach cascade or a realistic compressor stage, but the principles are expected to be similar. Thus, future work will involve these other cases.

## Figures and Tables

**Table 1:** Geometrical parameters.

Parameter	Numerical Value
*b* (mm)	128.23
*h* (mm)	160
*t* (mm)	80.14
*b/t*	1.6
*h/b*	1.25
*B* (mm)	125.87
*β_1P_* (°)	−32
*β_2P_* (°)	10
*θ* (°)	42
*β_b_* (°)	−11

**Table 2:** Parametric groove schemes.

Scheme	W (mm)	H (mm)
Case 1	3.0	2.5
Case 2	1.5	1.0
Case 3	2.0	1.0
Case 4	2.5	1.0
Case 5	5.5	1.0
Case 6	2.5	0.8
Case 7	5.5	0.8
Case 8	2.5	0.5
Case 9	5.5	0.5

## References

[1] Gbadebo S.A., Cumpsty N.A., Hynes T.P. (2005). Three-Dimensional Separations in Axial Compressors. J. Turbomach..

[2] Lei V.M., Spakovszky Z.S. (2008). A Criterion for Axial Compressor Hub-Corner Stall. J. Turbomach..

[3] Vinuesa R., Schlatter P., Nagib H.M. (2018). Secondary flow in Turbulent Ducts with Increasing Aspect Ratio. Phys. Rev. Fluids.

[4] Denton J.D. (1993). Loss Mechanisms in Turbomachinery. J. Turbomach..

[5] de Haller P. (1953). Das verhalten yon tragflfigelgittern in axialverdichtern und im windkanal. BWK.

[6] Horlock J.H., Louis J.F., Percival P.M.E., Lakshminarayana B. (1966). Wall Stall in Compressor Cascades. J. Basic Eng..

[7] Joslyn H., Dring R. (1985). Axial Compress or Stator Aerodynamics. ASME J. Eng. Gas Turbines Power.

[8] Dong Y., Gallimore S.J., Hodson H.P. (1987). Three-Dimensional Flow and Loss Reduction in Axial Compressor. ASME J. Turbomach..

[9] Schulz H.D., Gallus H.D. (1988). Experimental Investigations of the Three-Dimensional Flows in an Annular Compressor Cascade. ASME J. Turbomach..

[10] Wang H., Lin D., Su X., Yuan X. (2017). Entropy Analysis of the Interaction between the Corner Sparation and Wakes in a Compressor Cascade. Entropy.

[11] Breugelmans F.A.E., Carels Y., Demut M. (1984). Influence of Dihedral on the Secondary Flow in a Two Dimensional Compressor Cascade. ASME J. Eng. Gas Turbines Power.

[12] Weing old H.D., Neuber T.R.J., Behlke R.F., Potter G.E. (1997). Bowed stator: An Example of CFD Applied to Improve Multistage Compressor Efficiency. ASME J. Turbomach..

[13] Fischer A. Performance of Strongly Bowed Stators in a 4-Stage High Speed Compressor. Proceedings of the ASME Turbo Expo 2003, Collocated with the 2003 International Joint Power Generation Conference.

[14] Tweedt D.L., Okiishi T.H., Hathaway M.D. (1986). Stator Endwall Leading-Edge Sweep and Hub Shroud Influence on Compressor Performance. J. Turbomach..

[15] Wadia A.R. (2005). Some Advances in Fan and Compressor Aero at GE Aircraft Engines.

[16] Gummer V., Wenger U., Kau H.P. (2001). Using Sweep and Dihedral to Control Three-Dimensional Flow in Transonic Stators of AxialCompressor. J. Turbomach..

[17] Hartland J.C., Smith G. (1998). Non-Axisymmetric Endwall Profiling in a Turbine Rotor Blade. Proceedings of the ASME 1998 International Gas Turbine and Aeroengine Congress and Exhibition.

[18] Dorfner C., Nicke E., Voss C. (2007). Axissymmetric Profiled Endwall Design Using Multiobjective Optimization Linked with 3D Rans Flow Simulations. Turbo Expo 2007: Power for Land, Sea, and Air.

[19] Harvey N.W., Offord T.P. (2008). Some Effects of Non-Axisymmetric End Wall Profiling on Axial Flow Compressor Aerodynamics: Part II, Multi-Stage HPC CFD study. ASME Turbo Expo 2008: Power for Land, Sea, and Air.

[20] Merchant A.A. (1999). Design and Analysis of Axial Aspirated Compressor Stage. Ph.D. Thesis.

[21] Gbadebo S.A., Cumpsty N.A., Hynes T.P. (2008). Control of Three-Dimensional Separations in Axial Compressors by Tailored Boundary Layer Suction. J. Turbomach..

[22] Roth J.R., Dai X. The Physics and Phenomenology of Paraelectric one Atmosphere Uniform Glow charge Plasma Actuarors for Aerodynamic flow Control. Proceedings of the 43rd AIAA Aerospace Sciences Meeting and Exhibit.

[23] Rethmel C., Little J., Takashima K. Flow Separation Control Over an Airfoil with Nanosecond Pluse Driven DBD Plasma Actuators. Proceedings of the 49th AIAA Aerospace Sciences Meeting Including the New Horizons Forum and Aerospace Exposition.

[24] Jim’enez J. Wall friction and the structure of near-Wall turbulence. Proceedings of the 11th Australasian Fluid Mechanics Conference.

[25] Suzuki Y., Kasagi N. (1994). Turbulent drag reduction mechanism above a riblet surface. AIAA J..

[26] Akinlade O.G. (2005). Effects of Surface Roughness on the Low Charachteristics in a Turbulant Boundary Layer. Ph.D. Thesis.

[27] Luchini P., Manzo F., Pozzi A. (1991). Resistance of a grooved surface to parallel flow and cross-Flow. J. Fluid Mech..

[28] Ricardo G.M., Javier J. (2011). Hydrodynamic stability and breakdown ofthe viscous regime over riblets. J. Fluid Mech..

[29] Miao X., Zhang Q., Atkin C., Sun Z. End-Wall Secondary Flow Control Using Engineered Residual Surface Structure. Proceedings of the ASME Turbo Expo 2016: Turbomachinery Technical Conference and Exposition. American Society of Mechanical Engineers Digital Collection.

[30] Miao X., Zhang Q., Wang L., Jiang H., Qi H. (2015). Application of Riblets on Turbine Blade End-Wall Secondary Flow Control. J. Propuls. Power.

[31] Khader M.A., Saym A.I. (2017). Effect of end-Wall riblets on radial turbine Performance. IOP Conference Series: Materials Science and Engineering.

[32] Oehlert K., Seume J. (2006). Exploratory Experiments on Machined Riblets on Compressor Blades. ASME Fluids Engineering Division.

[33] Hergt A., Hage W., Grund S., Steinert W., Terhorst M., Schongen F., Wilke Y. (2015). Riblet Application in Compressors: Toward Efficient Blade Design. J. Turbomach..

[34] Dorfner C., Hergt A. (2011). Advanced Nonaxisymmetric Endwall Contouring for Axial Compressors by Generating an Aerodynamic Separator—Part I: Principal Cascade Design and Compressor Application. J. Turbomach..

[35] Li J., Ji L., Yi W. The Use of Blended Blade and End Wall in Compressor Cascade: Optimization Design and Flow Mechanism. Proceedings of the ASME Turbo Expo 2018: Turbomachinery Technical Conference and Exposition.

[36] Li J., Ji L., Yi W. Experimental and Numerical Investigation on the Aerodynamic Performance of a Compressor Cascade Using Blended Blade and End Wall. Proceedings of the ASME Turbo Expo 2017: Turbomachinery Technical Conference and Exposition.

[37] Gmelin C., Zander V. (2012). Active Flow Control Concepts on a Highly Loaded Subsonic Compressor Cascade: Re’sume’ of Experimental and Numerical Results. J. Turbomach..

[38] Mao X., Liu B., Tang T. (2018). Effect of casing aspiration on the tip leakage flow in the axial flow compressor cascade. Proc. Inst. Mech. Eng. Part A.

[39] Spalart P.R., Allmaras S.R. A One-Equation turbulence model for aerodynamic flows. Proceedings of the 30th Aerospace Sciences Meeting and Exhibit.

[40] Numeca International (2016). User Guide for FINE™/Turbo.

